# Production and characterization of bioemulsifier by *Parapedobacter indicus*

**DOI:** 10.3389/fmicb.2023.1111135

**Published:** 2023-02-16

**Authors:** Anushka Devale, Rupali Sawant, Karishma Pardesi, Kahkashan Perveen, Mehrun NIsha Khanam, Yogesh Shouche, Shilpa Mujumdar

**Affiliations:** ^1^Department of Microbiology, P.E.S. Modern College of Arts, Science and Commerce (Autonomous), Pune, India; ^2^School of Arts and Sciences, Azim Premji University, Bengaluru, India; ^3^Department of Microbiology, Savitribai Phule Pune University, Pune, India; ^4^Department of Botany and Microbiology, College of Science, King Saud University, Riyadh, Saudi Arabia; ^5^School of Biological Sciences, College of Natural Sciences, Seoul National University, Seoul, Republic of Korea

**Keywords:** bioemulsifier, *Parapedobacter*, heavy metal resistance, bioremediation, BS/BE

## Abstract

The current study evaluated *Parapedobacter indicus* MCC 2546 for its potential to produce a bioemulsifier (BE). Screening methods performed for BE production by *P. indicus* MCC 2546 showed good lipase activity, positive drop collapse test, and oil-spreading activity. Furthermore, it showed maximum emulsification activity (225 EU/ml) and emulsification index (E_24_ 50%) at 37°C in Luria Bertani broth at 72 h with olive oil as a substrate. The optimal pH and NaCl concentration for maximum emulsification activity were 7 and 1%, respectively. *P. indicus* MCC 2546 lowered the surface tension of the culture medium from 59.65 to 50.42 ± 0.78 mN/m. BE produced was composed of 70% protein and 30% carbohydrate, which showed the protein–polysaccharide nature of the BE. Furthermore, Fourier transform infrared spectroscopy analysis confirmed the same. *P. indicus* MCC 2546 showed a catecholate type of siderophore production. This is the first report on BE and siderophore production by the genus *Parapedobacter.*

## 1. Introduction

Biosurfactants (BS) and bioemulsifiers (BE) are amphiphilic surface active compounds (SACs). They are further classified based on their molecular weights and properties (Ron and Rosenberg, 2001; Bodour and Maier, 2003; Franzetti et al., 2010). BS are low-molecular-weight SACs of simple constituents such as amino acids, sugars, and fatty acids. BEs are high-molecular-weight SACs comprising complex mixtures of biomolecules such as proteins, lipoproteins, heteropolysaccharides, and lipopolysaccharides (Markande and Nerurkar, 2019). Both have surface active properties leading to surface and interfacial tension reduction, but BE is more effective in stabilizing oil-in-water emulsions (Uzoigwe et al., 2015).

Biosurfactants and BE have gained increased importance in recent years due to their potential applications in various industries such as pharmaceutical, agriculture, food, medicine, textile, and oil industry (Kumar et al., 2021; Raj et al., 2021; Dong et al., 2022; Pardhi et al., 2022; Thraeib et al., 2022). They play a key role in industries by exhibiting different properties such as surface and interfacial tension reduction, wetting agent, oil dispersion, cleansing agent, and emulsification (da Silva Lira et al., 2022). The Amendment of BS/BE producers in the bioremediation of polycyclic aromatic hydrocarbons and heavy metals is considered a modern and popular technique to deal with pollution-related issues. To date, chemical surfactants such as detergents and emulsifiers have been widely used in heavy metal and PAH removal. The surfactants employ two strategies: immobilization of heavy metal in the solid matrix, which is bound in the soil, or the mobility of heavy metal and PAH to the liquid phase by solubilization and desorption (Singh et al., 2007; Sarubbo et al., 2018). This further makes it available for biological degradation or assimilation by indigenous microorganisms. Various chemical surfactants are available, but they pose a severe problem of environmental pollution and toxicity problem. Due to the increase in PAH and heavy metal pollution, there is a need for the substitution of chemical surfactants. BS/BE have many advantages over chemical surfactants such as low toxicity, high specificity, biocompatibility, biodegradability, and tolerance to extreme environmental conditions (Juwarkar et al., 2008; Monteiro et al., 2010; Sarubbo et al., 2018; Ravindran et al., 2020; Ali et al., 2022). Although BS/BE production is challenging, current extraction and purification methods such as foam fractionation, ultrafiltration, thin-layer chromatography, high-performance liquid chromatography, and gas chromatography-mass spectrometry made it possible as they are inexpensive (Jimoh and Lin, 2019; Arora et al., 2021; da Silva Lira et al., 2022).

Biosurfactants have many prospective applications and attractive features. Hence, their global demand is increasing periodically (Twigg et al., 2021). Numerous microbes have been reported to produce BS/BE. The microbial genera extensively studied for BS/BE production are *Bacillus*, *Pseudomonas*, *Halomonas*, *Acinetobacter, Serratia, Candida, Penicillium, Yarrowia, Torulopsis, Aspergillus, Pseudozyma, Saccharomyces, Trichosporon*, and *Rhodotorula* (Bodour and Maier, 2003; Bonilla et al., 2005; Fontes et al., 2010; Satpute et al., 2010; Bhairav et al., 2013; Bakhshi et al., 2018; Płaza and Achal, 2020; Baskaran et al., 2021; Guergouri et al., 2022). The SACs produced by only a few of these mentioned microbial genera are comprehensively studied, well-characterized, and commercialized in the market (Franzetti et al., 2010). Singh et al. (2019) have stated that by 2023 the global BS/BE market revenue generation will reach US $2.6 billion, and the demand for BS/BE will be 540 kilotons by 2024. This indicates tremendous demand for BS and BE in the global market and needs to be addressed soon. To cope with the market demand for BS and BE, novel microbial resources producing novel BS/BE with better features must be discovered and studied in detail (Hasani zadeh et al., 2018).

Siderophores are small molecular weight ferric-specific ligands produced by various microorganisms for their iron nutrition (Sayyed and Chincholkar, 2011). Although siderophores are ferric-specific ligands, they can also bind multiple other metal ions in the environment (Schwyn and Neiland, 1987; Payne, 1994). The present research aimed to study BE production by *Parapedobacter indicus* (MCC 2546) and explore its possible application in bioremediation.

## 2. Materials and methods

### 2.1. Bacterial strain

The bacterial-type strain *P. indicus* (DSM 28470 ^T^ = MCC 2546^T^), used in the current study, was provided by the National Centre for Microbial Resource (NCMR), Pune, Maharashtra, India. The culture was initially isolated from a hexachlorocyclohexane (HCH) dumpsite in Lucknow, India (Kumar et al., 2015). The culture was preserved in glycerol stocks, while the working culture was maintained on Luria agar slants and was cultivated in Luria Bertani (LB) broth for all further experiments.

### 2.2. Screening for BE production

*Parapedobacter indicus* was screened for BE production based on various screening methods, namely, hemolytic activity (HA), modified drop collapse (MDC) method, parafilm M test, lipase activity, oil spread method (OSM), blue agar plate (BAP) method, hydrocarbon overlay agar (HOA) method, emulsification assay (EA), emulsification index (EI), and tensiometric measurement by pendant drop technique (Satpute et al., 2010; Thavasi et al., 2011).

#### 2.2.1. HOA method

Sterile LB agar plates were coated with 20 μl hydrocarbons, olive oil, and Pennzoil. Hydrocarbon-coated LB plates were spot inoculated with 48 h grown bacterial culture and incubated at 28°C for 72 h. If the colony produced emulsified halos, it was recorded as a positive test (Ali et al., 2021).

#### 2.2.2. HA

The human blood agar plates were spot inoculated with 72 h grown culture of *P. indicus* and incubated at 28°C for 48–72 h. Incubated plates were observed for a halo zone around the spotted colonies (Shah et al., 2016; Nogueira et al., 2020).

#### 2.2.3. MDC method

Each well of the 96-well microtiter plate was thinly coated with olive oil and Pennzoil, and 72 h grown culture supernatant (5 μl) was placed in the center of the well. A drop of the uninoculated medium was used as a control (Zarinviarsagh et al., 2017). The drop was observed for a minute. If the drop destabilized within a minute, it was recorded as a positive MDC test.

#### 2.2.4. Parafilm M test

Twenty microliter aliquots of 72-h grown culture supernatant were placed on a strip of parafilm, and the drop’s shape was observed for 1 min. If the drop destabilized within a minute, it was recorded as a positive result. A drop of the uninoculated medium was used as a control (Patel and Patel, 2020).

#### 2.2.5. Lipase activity

Forty-eight hours old bacterial culture was spot inoculated on sterile tributyrin agar (TBA) plates and incubated at 28°C for 72 h. A zone of clearance around the colony was observed, and the zone diameter was measured in mm (de Carvalho-Gonçalves and Gorlach-Lira, 2018).

#### 2.2.6. Oil-spreading method (OSM)

A thin uniform layer of 10 μl of olive oil was added to 40 ml of distilled water in a Petri dish. Twenty microliters of 72-h grown culture supernatant were placed on the oil layer. The diameter of the oil displacement zone was also recorded (Walter et al., 2010). If the oil is displaced and a clear zone is formed, it is considered a positive oil-spreading test.

#### 2.2.7. BAP method

Sterile BAPs [0.2 g of cetyltrimethylammonium bromide (CTAB), 0.005 g of methylene blue, and 15 g of agar to 1 L LB medium] were spot inoculated with 72 h grown culture of *P. indicus*. The plates were observed for dark blue halos around the colony after 72 h of incubation at 28°C, which indicates a positive BAP test (Hamzah et al., 2020).

#### 2.2.8. Emulsification assay (EA)

*Parapedobacter indicus* was grown for 72 h in the LB medium. Microbial cells were separated by centrifugation at 10,000 rpm for 10 min at room temperature. Six-milliliter supernatant was mixed with 1 ml oil/hydrocarbon and vortexed vigorously for 2 min. The mixture was left undisturbed for 1 h to separate the aqueous and oil phase. The absorbance of the aqueous phase was noted at 400 nm. The blank was prepared by replacing the cell-free supernatant with a sterile LB medium. The absorbance of 0.010 units at 400 nm multiplied by the dilution factor (if any) was considered as one unit of emulsification activity (EA) per ml (EU/ml) (Maniyar et al., 2011; Dharmadevi et al., 2022).

#### 2.2.9. Emulsification index (EI)

Emulsification index was measured by mixing 2 ml of cell-free supernatant with 2 ml of hydrocarbon/oil, vortexed at high speed for 2 min. The sample was left undisturbed for 24 h at 25°C. The EI was expressed in percentage. The EI was calculated by using the following formula:


$$E\,I\,\left(\text{E}\,24\%\right)=\frac{H\,e\,i\,g\,h\,t\,o\,f\,t\,h\,e\,e\,m\,u\,l\,s\,i\,o\,n\,l\,a\,y\,e\,r}{\mathrm{The}\,\mathrm{total}\,\mathrm{height}\,\mathrm{of}\,\mathrm{the}\,\mathrm{liquid}\,\mathrm{column}}\times 100$$

([Bibr B75][Bibr B86]; Silva et al., 2022).

#### 2.2.10. Surface tension (ST) measurement

ST reduction is one of the important properties of all surfactants. Cell-free supernatant of the 72-h-old culture was used to determine ST using the pendant drop method. The culture supernatant drop was hung on a dosing needle to capture the images. The results were expressed from the average of 40 ST-values in mN/m with the help of a computerized image processing system (Walter et al., 2010). ST readings were taken in triplicates.

### 2.3. Effect of different oils and hydrocarbons on BE production

The effect of different oils and hydrocarbons on BE production was measured using different oils/hydrocarbons as substrates. Different oils used were olive oil, sunflower oil, engine oil (Pennzoil and Castor), and groundnut oil. Different hydrocarbons used were diesel, petrol, kerosene, toluene, and xylene. Each flask was inoculated with *P. indicus* in LB broth and incubated at 28°C at 90 rpm. For each oil and hydrocarbon, EA and EI were measured separately every 24 h (Amodu et al., 2014; Maia et al., 2018).

### 2.4. Optimization of growth medium for BE production

The effect of various media on BE production by *P. indicus* was studied. The media used were Bushnell Hass medium (BH) + 1% glucose, LB medium, Minimal Salt medium (MSM) +1% glucose, and M9 medium + 1% glucose. All these media were separately inoculated with *P. indicus*. Flasks were incubated at 28°C at 90 rpm. After each 24 h, EA and E were measured independently to estimate the emulsification potential of the culture using olive oil.

### 2.5. Time course of BE production by *P. indicus*

The time course for the production of BE was investigated by inoculating *P. indicus* in an LB medium. The flasks were incubated at 28°C at 90 rpm. BE production was measured every 24 h and expressed as EA and EI. The biomass production was also recorded after each 24 h interval by measuring O.D. at 600 nm (Gudiña et al., 2015).

### 2.6. Effect of inoculum size on BE production

To evaluate the effect of inoculum size on BE production, 100 ml of LB medium was inoculated with 0.5, 1, 2, and 3% v/v inoculum of *P. indicus* (OD_600__*nm*_ = 0.1). The optical density of the culture medium was measured at 600 nm up to 120 h. EA and EI of cell-free supernatant were measured after each 24 h interval up to 120 h (Yanti et al., 2018).

### 2.7. Optimization of physiological parameters for BE production

The BE production ability of *P. indicus* was studied at different growth conditions, such as temperature, pH, and NaCl concentration. Temperature ranges such as 28, 37, and 40°C were studied to find the effect of temperature on BE production. The optimum pH was determined by monitoring production in buffered LB media of pH ranging from 5, 6, 7, 8, and 9 at 37°C. Optimum NaCl concentration was investigated by inoculating *P. indicus* in LB medium with 0.5, 1, 2, 3, and 4 g% (w/v) NaCl at 37°C. The inoculum size used for all the above experiments was 1% (v/v). EA and EI measured the BE production ability for 48, 72, and 96 h at 90 rpm (Saikia et al., 2012).

### 2.8. Effect of carbon: Nitrogen (C:N) ratios on BE production

The effect of carbon and nitrogen sources on the production of BE by *P. indicus* was studied using LB media supplemented with different C:N ratios such as 1:1, 1:2, and 2:1. Glucose and ammonium sulfate were used as carbon and nitrogen sources, respectively. The experiment was carried out at 37°C at 90 rpm. The BE production was observed at 48, 72, and 96 h of incubation, and EA and EI were recorded.

### 2.9. Extraction and partial purification of BE

For extraction of BE, 72 h incubated bacterial culture supernatant was used. One part of the culture supernatant was mixed with three parts of chilled ethanol for precipitation, which was followed by overnight incubation at 4°C (Adetunji and Olaniran, 2019). The precipitated BE was dialyzed in a cellulose tube membrane (molecular weight cut off 12,000–14,000 Daltons; Hi-media, India) for 48 h against distilled water, followed by lyophilization. The weight of the lyophilized product was measured.

### 2.10. Chemical composition of BE

The chemical composition of BE was studied using standard methods (Hyder, 2015). The phenol–sulfuric acid method determined the total carbohydrate content using D-glucose as a standard (Dubois et al., 1956). Protein content was measured using the Folin–Lowry method with bovine serum albumin as a standard (Lowry et al., 1951).

### 2.11. Fourier transform infrared (FTIR) spectroscopic analysis

Functional groups of the BE sample were elucidated by FTIR spectroscopic study. A total of 1 mg powder of BE was used for FTIR. The FTIR spectrum was generated in the wave number range of 3,500–500 cm^–1^ using an infrared spectrometer (Platinum ATR, ALPHA II, Bruker, Germany) at 25°C.

### 2.12. Siderophore production and heavy metal resistance

Siderophore production was estimated to determine the ability of *P. indicus* as plant growth-promoting rhizobacteria. Arnow and Csáky assays were conducted to determine the catechol and hydroxamate type of siderophore.

#### 2.12.1. Arnow’s assay

The cell-free supernatant of 72 h grown culture of *P. indicus* was obtained. In 3 ml of the cell-free supernatant, 0.3 ml of 5 N HCl, 1.5 ml of Arnow’s reagent (10 g NaNO_2_, 10 g Na_2_MoO_4_.2H_2_O dissolved in 50 ml distilled water), and 0.3 ml of 10 N NaOH were added. These were incubated for 10 min, and the development of pink color was observed (Ghazy and El-Nahrawy, 2021).

#### 2.12.2. Csáky assay

A total of 1 ml of 72 h grown cell-free supernatant and 1 ml of 6M H_2_SO_4_ were mixed and autoclaved for 30 min at 121°C. The mixture was allowed to cool. In the mix, 3 ml of sodium acetate (35%), 1 ml of sulfanilic acid (1% in 30% acetic acid), and 0.5 ml of iodine solution (1.3% in 30% acetic acid) were added. After 5 min of incubation at 25°C, excess iodine removal was carried out using 1 ml of trisodium arsenite (2% w/v). A total of 1 mL of α-naphthylamine (0.3% w/v) was prepared in acetic acid (30% v/v) and added to the solution, resulting in a change of color from orange to red (Ghazy and El-Nahrawy, 2021).

#### 2.12.3. Detection of heavy metal resistance

Heavy metal resistance of *P. indicus* was observed by determining its ability to grow on nutrient agar plates supplemented with different heavy metals separately. Each nutrient agar plate contained 30 mg/L of a heavy metal salt such as cobaltous chloride, mercuric chloride, cadmium chloride, lead chloride, and nickel chloride. Plates were inoculated with *P. indicus* and incubated at 37 °C. Growth was observed for 24–72 h (Mustapha and Halimoon, 2015).

## 3. Results

### 3.1. Production of BE by *P. indicus*

Screening methods such as lipase activity were performed to observe the strain’s lipolytic activity. A zone of clearance with 8 mm ± 0 diameter around the spotted colony on TBA plates was observed. It was confirmed that *P. indicus* has lipase activity. The OSM was used to find the surface activity of BS/BE produced by *P. indicus.* We found that BS/BE produced by *P. indicus* showed an olive oil-spreading diameter of 0.63 cm ± 0.12. EA and EI were carried out to check BS/BE production ability using olive oil. The EA and EI obtained were 173.5 EU/ml ± 6.5 and 51.56% ± 0.5, respectively. These positive results confirmed that strain *P. indicus* (MCC 2546) could produce BE/BS. This strain also showed a positive test for the MDC method and parafilm M test. Results of HOA, HA, and BAP were found to be negative (Table 1).

**TABLE 1:** Preliminary assessment of BS/BE production by *Parapedobacter indicus*.

BS/BE production test	Result
1. Hydrocarbon overlay agar (HOA) method	*-*
2. Hemolytic activity (HA)	*-*
3. Blue agar plate (BAP) method	*-*
4. Modified drop collapse (MDC) method and Parafilm M test	+
5. Lipase activity (TBA)	8 ± 0 (mm)
6. Oil spread method (OSM)	0.63 ± 0.12 (cm)
**7. Emulsification assay (EA: EU/ml)**	
Olive oil	**173.5 ± 6.5**
Sunflower oil	**160.5 ± 4.5**
Groundnut oil	**175 ± 25**
Pennzoil	123.93 ± 6.07
Diesel	37.27 ± 3.03
Castor engine oil	44.145 ± 3.35
Toluene	20.685 ± 0.91
Kerosene	32 ± 2
Xylene	18.3 ± 3.69
**8. Emulsification index (EI: E_24_ %)**	
Olive oil	**51.56 ± 0.5**
Sunflower oil	**46.88 ± 0**
Groundnut oil	**53.13 ± 2**
Pennzoil	35.94 ± 00.5
Diesel	31.25 ± 0
Castor engine oil	39.06 ± 00.5
Toluene	28.13 ± 1
Kerosene	21.88 ± 0
Xylene	21.88 ± 0
9. Tensiometeric measurement	50.42 ± 0.78 mN/m

−, negative test.

+, positive test. O.S.M., oil-spreading diameter in mm.

Lipase activity, diameter of the clear zone in mm.

EI > 40% and EA, highlighted in bold to denote high emulsification activity.

Quantitative results represented as mean ± S.D. of at least duplicate experiments.

To quantify and significantly confirm BS or BE production, a culture medium’s ST reduction was measured by tensiometer using the pendant drop method. It was found that BE produced by the *P. indicus* strain could reduce the ST of the culture medium from 59.65 to 50.42 ± 0.78 mN/m. This confirmed the BE nature of the SAC produced by strain *P. indicus.*

### 3.2. Effect of different oils and hydrocarbons

Amongst different oils tested as a substrate for EA, olive oil and sunflower oil showed maximum EA and EI. The values for EA recorded for olive oil and groundnut oil were 173.5 EU/ml ± 6.5 and 175 EU/ml ± 25, respectively. While the values noted for EI of olive oil and groundnut oil were 51.56% ± 0.5 and 53.13% ± 2, respectively. Sunflower oil also showed good EA, i.e., 160.5 EU/ml ± 4.5, and EI, i.e., 46.88%. Even though groundnut oil showed maximum EA and EI, it could not be used for further studies as effective emulsion was also observed in control tubes.

### 3.3. Time course and inoculum size for BE production

**FIGURE 1:**
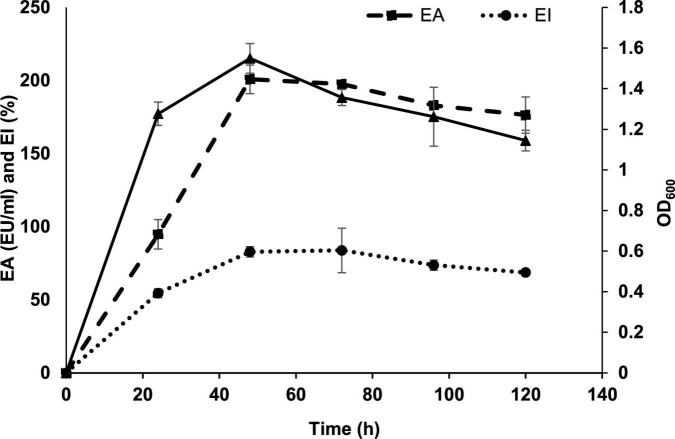
Time course of BE production by *Parapedobacter indicus.*

**FIGURE 2:**
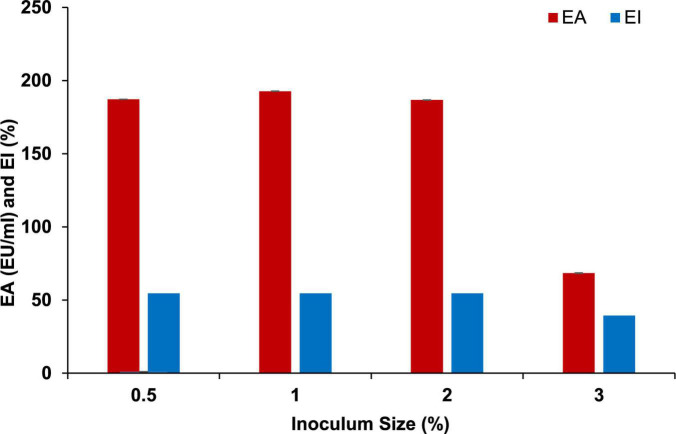
Effect of inoculum size on BE production by *Parapedobacter indicus.*

The growth kinetics of *P. indicus* showed maximum EA, index, and growth at 72 h. Figure 1 shows the time course of BE production by *P. indicus.* Inoculum size and nutrients in the medium are very important to achieve maximum production of the desired metabolite. To obtain optimum BE production, we considered four different inoculum sizes such as 0.5, 1, 2, and 3% v/v. We found that 1% (v/v) inoculum size was optimum to achieve maximum BE production by *P. indicus* (Figure 2).

### 3.4. Optimization of growth conditions

**FIGURE 3:**
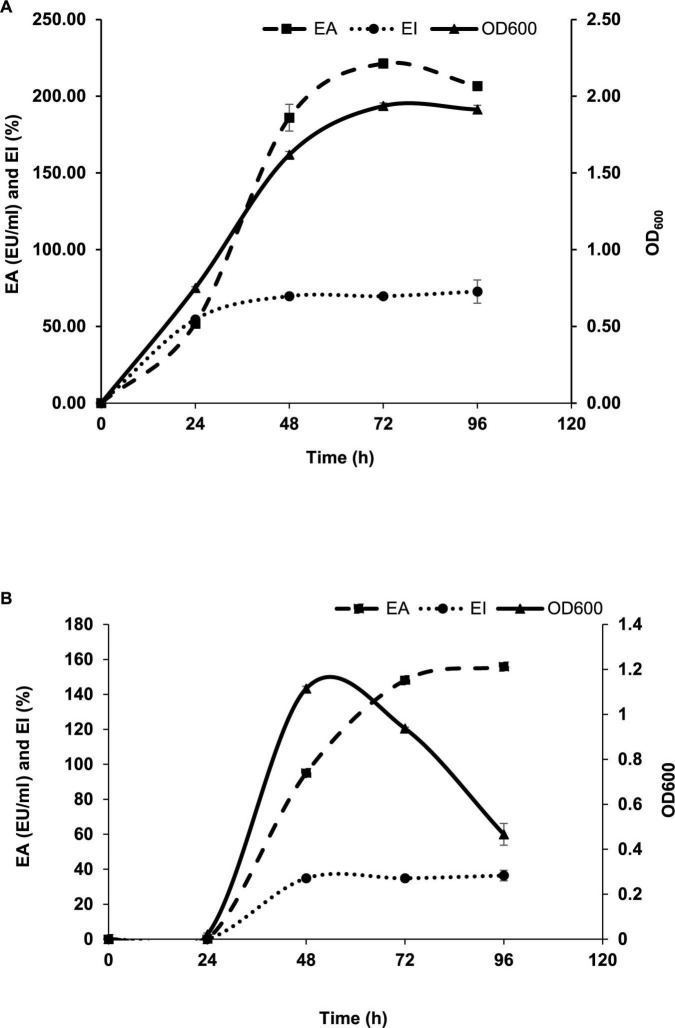
**(A)** Effect of LB medium on growth and BE production ability of *Parapedobacter indicus.* **(B)** Effect of MSM amended with 1% glucose medium on the growth and BE production ability of *Parapedobacter indicus.*

**FIGURE 4:**
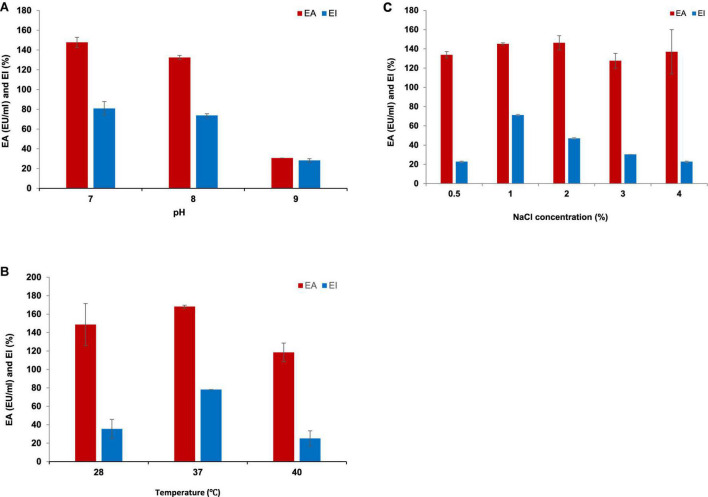
**(A)** Effect of pH on BE production by *Parapedobacter indicus.* **(B)** Effect of temperature on BE production by *Parapedobacter indicus.* **(C)** Effect of NaCl concentration on BE production by *Parapedobacter indicus.*

**FIGURE 5:**
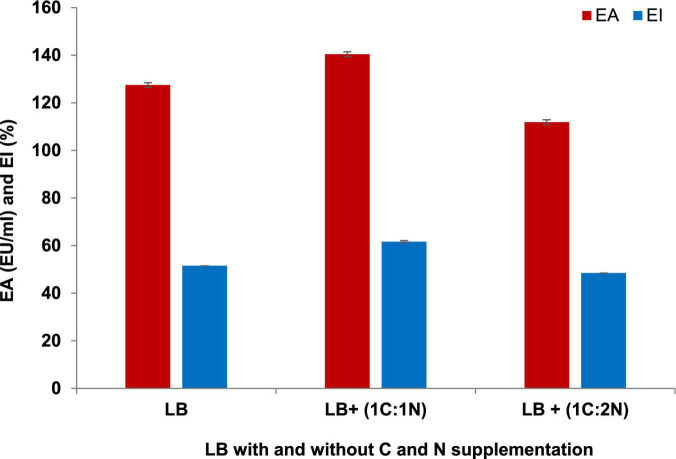
Effect of C/N ratio on BE production by *Parapedobacter indicus.*

Luria Bertani broth was the most suitable medium for the BE production and growth of *P. indicus.* LB’s maximum EA and EI were 221.30 EU/ml and 51.56%, respectively. Figures 3A, B show the effect of LB and M.S.M. media on the growth of *P. indicus* and its BE production ability, respectively. It was observed that *P. indicus* could not grow in BH and M9 media. Environmental factors such as temperature, pH, and salt concentration play important roles in BE production and activity. The optimum temperature recorded for maximum BE production was 37°C. In the case of pH, *P. indicus* could not grow at pH 5 and showed delayed growth at pH 6 with no EA. Among pH 7, 8, and 9, bacteria exhibited good EA at pH 7 and 8. However, maximum EA was recorded at pH 7. A study was carried out to check the effect of NaCl concentration on BE production. NaCl concentration of 1 and 2% (w/v) was found to be most effective for BE production. Overall, temperature 37°C, pH 7, and 1% w/v NaCl concentration were optimum for maximum BE production (Figures 4A–C). The optimal C:N ratio for the production of BE in LB medium was 1:1 (Figure 5). The carbon and nitrogen sources were glucose (1 g/100 ml) and ammonium sulfate (1 g/100 ml). It was also observed that *P. indicus* could not grow in an LB medium supplemented with a 2:2 ratio of carbon and nitrogen.

### 3.5. Extraction, partial purification, and chemical composition

**FIGURE 6:**
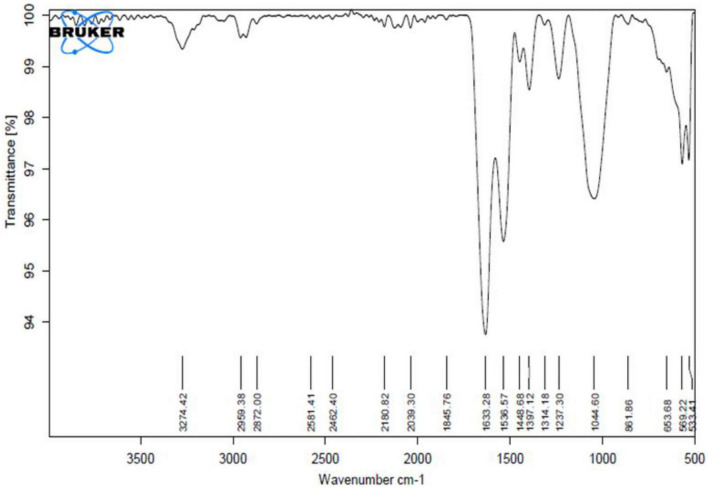
FTIR spectra of BE produced by *Parapedobacter indicus.*

After extraction with ethanol and partial purification, the BE yield obtained was 0.321 g/L. The BE obtained was off-white and had amorphous powdery nature after lyophilization. It was as light as cotton in weight and easily soluble in water at room temperature. The BE obtained has 70% protein and 30% polysaccharide, which confirms its “protein–polysaccharide” nature. The chemical composition and other properties of the BE are given in Table 2. The FTIR spectrum obtained for BE showed bands at 3,274 cm^–1^, which revealed the presence of hydroxyl groups indicating the presence of carbohydrates and carboxylic acids (Nogueira et al., 2020). In addition, the distinct absorption band at 1,633^–1^ and 1,536 cm^–1^ C = O showed the stretching mode of the C–O–N bond of acetamido groups of *N*-acetylated sugars and the N–H bend of primary amines. Moreover, a band at 1,632 cm^–1^ corresponds to the N–H bend. Absorption bands at 1,448 and 1,314 cm^–1^ correspond to C–H stretch of aliphatic amines. Furthermore, absorption bands at 1,044 cm^–1^ indicate the presence of aliphatic amines. The FTIR study suggested that the BE is composed mainly of proteins and polysaccharides. Based on the above results, BE was preliminarily identified as a protein–polysaccharide complex (Figure 6).

**TABLE 2:** Properties of bioemulsifier produced by *Parapedobacter indicus.*

Property	Description
Color	Off-white
Yield	0.321 g/L
Nature	Off white, cottony amorphous powder
Chemical composition	Protein: 70% Carbohydrate: 30%
Solubility	Completely soluble in water (at Room temperature (25–28^°^C)

### 3.6. Siderophore production and heavy metal resistance by *P. indicus*

*Parapedobacter indicus* was studied for both catechol and hydroxymate type of siderophore production. It was found that *P. indicus* showed a positive test for the catecholate type of siderophore only. Nutrient agar plates with different heavy metals were observed for growth for heavy metal resistance. *P. indicus* showed growth on nutrient agar plates containing 30 mg/L heavy metal salts such as cobaltous chloride, cadmium chloride, lead chloride, and nickel chloride, except mercuric chloride. It displayed the heavy metal resistance ability of *P. indicus.*

## 4. Discussion

*Parapedobacter indicus* MCC 2546 strain used in the present study was not previously studied for the production of BE or BS. To the best of our knowledge, there is no report available on the production of BE or BS from the genus *Parapedobacter*. Sayyed et al. (2015) and Tyagi et al. (2020) reported exopolysaccharides (EPS) production by *Parapedobacter* sp. ISTM3 and *Enterobacter* sp. RZS5, respectively, and explored their application in the biosorption of heavy metals. However, properties such as BE or BS production by the genus *Parapedobacter* have not been studied. Different screening methods were studied to investigate the ability of *P. indicus* for BE or BS production. The hydrocarbon overlaid agar (HOA) plate method is used to detect the hydrocarbon-degrading ability of the organisms. The HOA method exhibited negative results as the strain could not grow on HOA. This showed its inability to utilize tested hydrocarbons as the sole carbon source. Similar results were reported by Nayarisseri et al. (2018), which showed negative results for *Escherichia coli* and *Bacillus. E. coli* showed a negative test for toluene, and *Bacillus* showed a negative test for kerosene and benzene (Nayarisseri et al., 2018). The CTAB agar plate method is used to detect extracellular anionic surfactants. Colonies on the BAP did not show any halo, suggesting that the BS/BE produced by *P. indicus* was not anionic. Comparable results were reported by Varjani et al. (2014) by the 25 BS/BE-producing strains isolated from crude oil-contaminated soils from Gujrat, India (Varjani et al., 2014). Thavasi et al. (2011) stated that CTAB is harmful, prevents the growth of some microbes, and can be replaced by another cationic surfactant (Thavasi et al., 2011). The hemolysis test is the primary screening method to detect BE or BS production. A negative hemolysis test shown by *P. indicus* can be considered a positive feature as beta hemolysis is a predictive feature for pathogenic microbes. Mulligan (2005) recommended that BS or BE detection methods based on surface activity measurements should be preferred over the HA method (Mulligan, 2005). Similarly, Satpute et al. (2008) elaborated on different screening methods for selecting BS-producing bacteria. They stated that the bacteria that showed negative hemolysis or HOA test could have the potential for BS/BE production based on other screening methods (Satpute et al., 2008). Lipase activity for our strain showed positive results and recorded an 8 mm zone of clearance around the colony. Similar results were noted by Doshi et al. (2010) and Maniyar et al. (2011) for *Actinopolyspora* and *Streptomyces* strains isolated from garden soil. The MDC method is based on the principle that distilled water alone did not collapse on the oily surface but surfactant added in the same can spread the drop. The MDC method showed positive results for BE produced by *P. indicus.* Several reports state positive results for the drops collapse method for BS or are produced by different organisms (Tugrul and Cansunar, 2005; Varjani et al., 2014; Zhou et al., 2015). Patowary et al. (2017) recently found a potential strain of *Pseudomonas aeruginosa* PG1 to produce BS and showed a positive result for the drop collapse test. They observed that adding the culture broth of *P. aeruginosa* collapsed the drop of crude oil within 1 min, indicating BS’s presence in the culture medium. In the case of the parafilm M test, BE produced by *P. indicus* showed drop collapse within 1 min. Patel and Patel (2020) described similar results for yeast isolates, namely, Ky-48, Ky-53, Ky-54, Ky-84, Ky-46, and Ky-87. They found the highest activity by isolate Ky-46, followed by isolate Ky-86 (Patel and Patel, 2020). The OSM was essential to find the properties of BS or BE as a SAC. We have recorded positive results with an oil displacement diameter of 0.63 ± 0.12 cm, indicating the ability of *P. indicus* to produce BE. Similar results were noted by Thavasi et al. (2011), who worked on different screening methods for marine isolates. They found the oil-spreading diameter for their isolates in the range of 0.5–3.5 cm (Thavasi et al., 2011).

Surface tension reduction is considered to be the most important property of BS or BE. Strain *P. indicus* reduced the ST of culture media from 59.65 to 50.42 mN/m, indicating its ability to produce BE and not BS. Similar results were observed by Panjiar et al. (2015). They have isolated BE producer *Bacillus cereus* SP1035 from oil-contaminated soils. Researchers found that strain *B. cereus* SP1035 lowered the ST of the culture medium to 43.42 ± 0.03 mN/m (Panjiar et al., 2015). No significant reduction in ST was observed, but values were around the threshold of 40 mN/m (Olivera et al., 2003). This may be due to the production of polymeric BSs, which do not reduce ST considerably but have emulsification abilities (Płaza et al., 2006). Toledo et al. (2008) reported the production of BEs by three bacterial strains: *Bacillus subtilis* 28, *Alcaligenes faecalis* 212, and *Enterobacter* sp. 214. The bacterial culture supernatant of all strains exhibited good emulsifying activity (E_24_ up to 65% with various hydrocarbons such as n-octane, toluene, xylene, mineral light oil, heavy mineral oil, and crude oil). However, the ST reduction of culture media was observed up to 50 mN/m only. BE are responsible for emulsifying liquids without significant ST reduction of their growth medium (Uzoigwe et al., 2015). Previously, Gudiña et al. (2015) reported that the BE produced by *Paenibacillus* sp. #510 did not show any decrease in the ST (50.0 mN/m ± 0.6) of the culture medium with EI (performed using n-hexadecane) 62.1% ± 2.5. This observation supports that “bioemulsifiers do not reduce surface tension but exhibit good emulsification potential” (Gudiña et al., 2015). Recently, a yeast isolate, *Meyerozyma caribbica*, was reported by Bhaumik et al. (2020), which was able to produce a peptidoglycan-based BE. BE of *M. caribbica* exhibited 37% ± 0.185 E_24_ with diesel oil while ST reduction up to 49 mN/m only (Bhaumik et al., 2020). The *P. indicus* strain observed similar results as it showed good EA through EA (173.5 ± 6.5) and EI (51.56 ± 0.5) with olive oil. Still, they could not reduce the ST of culture broth significantly.

Different media were tested for optimization of the growth of *P. indicus*. It was previously observed and noted by Kumar et al. (2015) that optimal growth of *P. indicus* occurs in LB and R2A agar. We also observed that the LB medium was most suitable for the growth of *P. indicus* and BE production. Jagtap et al. (2010) used LB medium for the BE production from *Acinetobacter* sp. (Jagtap et al., 2010). Similarly, Peele and Kodali (2016) preferred LB medium to achieve maximum BE production by *Acinetobacter* spp. isolated from a marine environment. BE is known to be a secondary, growth-associated metabolite (Panjiar et al., 2015). Our results also revealed the same. The maximum BE production by *P. indicus* was recorded after 72 h incubation when the bacteria attained the stationary phase. It was observed that the production remained constant up to 120 h. Mujumdar (2005) reported the production of BE from *Acinetobacter baumannii* isolated from wheat rhizosphere soil. It was observed that maximum BE was produced in the stationary phase, i.e., at 72 h. Ilori and Amund (2001) reported peptide–glycolipid BE by *P. aeruginosa*. The BE production kinetics studies observed that the maximum BE produced in a stationary phase is at 120 h (Ilori and Amund, 2001). Studies on optimizing inoculum size for *P. indicus* showed that 1% was optimal for achieving maximum BE production. Furthermore, we observed that an increase in inoculum size showed a decrease in all parameters, namely, optical density, EA, and EI. This may be due to nutrient depletion for increased biomass metabolic activities. We did not find any report indicating a 1% optimal inoculum size for any microorganism that could produce BE. However, comparable results were noted by Motwali et al. (2021), who observed BS production by *Pseudomonas balearica* isolated from oil-contaminated sea waters. The optimal inoculum size for maximum BS production for this strain was 2% (v/v). Further rise in inoculum size resulted in a reduction in BS production (Motwali et al., 2021).

It is important to know BE’s ability and effectiveness at various environmental factors such as temperature, pH, and salinity to explore their suitable application/s (Banat et al., 2000). In the case of temperature optimization studies, several researchers reported 37°C as the optimum temperature to achieve maximum BE production (Patil and Chopade, 2001; Maniyar et al., 2011). Similarly, as per reports by Ainon et al. (2013), the hydrocarbon-degrading bacterium *P. aeruginosa* UKMP14T cultured in MSM produced maximum BS at a temperature of 37°C. Similar results were recorded by Peele and Kodali (2016) on *Acinetobacter* M6 isolated from the marine environment, where maximum BE production was noted at 37°C. In the case of pH optimization studies, *P. indicus* recorded maximum BE production at pH 7 (Peele and Kodali, 2016). It was also observed that it showed good BE production at pH 8 but reduced BE production at pH 9. Nitschke and Pastore (2006) reported good BE production at an alkaline pH range from 9 to 12. On the contrary, *B. cereus* was reported to produce BE, which is active only at pH 7 (Cooper and Goldenberg, 1987). In similar studies on pH optimization performed by Phetrong et al. (2008), the optimal pH for BE production by *Acinetobacter calcoaceticus* subsp. *Anitratus* was recorded at pH 7. Similarly, Panjiar et al. (2015) reported maximum BE production by *B. cereus* SP1035 at pH 7. NaCl salt concentration was studied to optimize BE production by *P. indicus*. We observed that 1% NaCl concentration was optimal for BE production by *P. indicus.* Jagtap et al. (2010) studied BE production by the *Acinetobacter lwoffii* TA38 strain isolated from healthy human skin, which exhibited maximum BE production in LB broth amended with 1% calcium chloride. Doshi et al. (2010) demonstrated maximum BE production by *Actinopolyspora* sp. A18. Their studies revealed that 2% NaCl concentration was best for maximum to BE production by *Actinopolyspora* sp. A18 (Doshi et al., 2010). It was also observed by Patil and Chopade (2001) that the BE from *Acinetobacter junii* SC14 was inhibited by CaCl_2_ and MgCl_2_.

The type and proportion of carbon and nitrogen in the medium also play a critical role in BS/BE production. In our studies, we have noted that the ratio of carbon and nitrogen in the medium affected the BE production by *P. indicus*. Recently, Bhaumik et al. (2020) studied the effect of the range of the C:N ratio on BE production by *M. caribbica*. Researchers kept the glucose concentration (50 g/L) constant in this investigation and varied ammonium sulfate concentration. The maximum BE production with a balance of cellular growth and metabolite production was achieved for 5 g/100 ml glucose and 0.3 g/100 ml ammonium sulfate (Bhaumik et al., 2020).

Chemical composition studies for BE produced by *P. indicus* showed that it is a complex of protein and polysaccharide containing 70% protein and 30% carbohydrate. This showed the rare composition of BE produced by *P. indicus* compared to previous reports. We find similar results with BE produced by Kokare et al. (2007), who studied BE production by marine *Streptomyces* sp. S1. The BE produced by this strain was composed of 82% of protein and 18% of carbohydrate. Although it showed similar composition to our BE, its protein-to-carbohydrate proportion varies. Patil and Chopade (2001) isolated *A. junii* SC 14 from healthy human skin. This BE is composed of a maximum percentage of protein (50.5%) and less percentage of carbohydrates (43%) and lipids (3.8%). They reported it as a proteoglycan type of BE. Steroid-transforming fungi, *C. lunata*, was reported for similar BE production, a heteropolymer composed of polysaccharides and protein without fat content. It comprises 25% protein, 48% carbohydrate, and 0% fat. If we compare the composition of BS produced by *P. indicus*, the percentage is exactly in contrast, i.e., 70% protein and 30% carbohydrate. Similar chemical composition was reported from well-known high mass to BE produced by *A. calcoaceticus* and *Acinetobacter radioresistens.* Emulsan and Alasan were reported as hetero-polysaccharide–protein complexes (Navon-Venezia et al., 1995), where polysaccharide composition is higher than protein. Markande et al. (2013) reported *Solibacillus silvestris* AM1 to produce similar results for thermostable glycoprotein. This BE constituted the maximum percentage of protein, i.e., 96.4%, and less percentage of carbohydrate, i.e., 3.6% (Markande et al., 2013). FTIR analysis for BE produced by *P. indicus* supported the chemical composition recorded by different chemical tests. BE produced showed absorption bands that displayed hydroxyl groups and amine groups, indicating the presence of protein and carbohydrates. Similar results were observed by Markande et al. (2013) for BE produced by strain *S. silvestris* AM1 (Markande et al., 2013). BS produced was a polysaccharide and protein complex with a higher polysaccharide percentage and protein content. Although BS produced by *P. indicus* showed a similar complex made up of polysaccharide and protein, the percentage of both the components is opposite compared to all the above BS produced by different microorganisms. There is no report available with a similar percentage of protein and carbohydrate to date. Even if the percentage of carbohydrates and protein is similar, such BE has also reported lipid content. For instance, Sarubbo et al. (2001) reported BE produced by *Candida lipolytica* in the presence of glucose as a carbon source consisting of 47% protein, 45% carbohydrate, and 5% lipids. Similar results on BE produced by *A. calcoaceticus* SM7 were observed. The BE produced was reported with 57.74% carbohydrate and 42.26% protein (Chamanrokh et al., 2008). These results are similar as there is no lipid content, but the protein percentage varies greatly compared to BE produced by *P. indicus*. Thus, the composition of BS produced by *P. indicus* becomes unusual.

*Parapedobacter indicus* exhibits siderophore production potential. Recently, similar results were observed by Valdez-Nuñez and Rivera-Jacinto (2022), where BS-producing *A. salavatliensis* and *A. gonensis* isolated from Peruvian hot springs showed siderophore production ability. Srivastava et al. (2022) reported siderophore production in *Pseudomonas monteilii* strain MN759447. Nithyapriya et al. (2021) reported siderophore production in *B. subtilis* and identified it as Bacillibactin. Production of catecholate and hydroxamate types of siderophore has been reported by Sayyed and Chincholkar (2006). Sayyed and Chincholkar (2011) and Valdez-Nuñez and Rivera-Jacinto (2022) reported the bioremediation of heavy metal-contaminated soil using siderophore-producing microorganisms. Akhtar et al. (2021) reported the potential of *B. cereus* to improve plant growth under chromium-contaminated soil.

In our research, heavy metal resistance was exhibited by *P. indicus* to cobalt, cadmium, lead, and nickel. This property can be further useful for removing heavy metal pollution from the soil. Gomaa and El-Meihy (2019) reported BS-producing *Citrobacter freundii* MG812314.1, which showed heavy metal resistance and removal of heavy metals such as aluminum, lead, zinc, cadmium, iron, copper, and manganese from wastewater. Combining siderophore and BE-producing bacteria (SPB) to remove heavy metal from contaminated soils is a modern practical approach (Garbisu and Alkorta, 2001; Newman and Reynolds, 2005; Wang et al., 2013). It is a known fact that bacterial iron-binding siderophores can bind other metal ions, hence, increasing trace nutrient availability to plants (Rajkumar et al., 2009, 2010). Thus, *P. indicus* can be considered a promising candidate in combination strategy for heavy metal-polluted soil bioremediation. Exploiting metal-resistant SPB is a promising approach for successful bioremediation and plant growth promotion.

## 5. Conclusion

*Parapedobacter indicus* MCC 2546 showed potential BE production. This is the first report of BE and siderophore production by the genus *Parapedobacter.* BE produced was a complex of protein and carbohydrate. Moreover, the strain showed heavy metal resistance and thus can be exploited in heavy metal bioremediation and agriculture. Furthermore, the role of *P. indicus* (*in vitro–in vivo*) for heavy metal removal with and without the combination of phytoremediation can be investigated.

## Data availability statement

The original contributions presented in this study are included in the article/supplementary material, further inquiries can be directed to the corresponding author.

## Author contributions

AD: conceptualization and writing—original draft. RS: draft proofreading. KPa: review of draft and provision of lyophilization facilities at the Department of Microbiology, Savitribai Phule Pune University. KPe and MK: writing, reviewing, and editing the revision. YS: assistance in procuring the cultures from NCMR. SM: study conception, design, analysis, and review. All authors contributed to the article and approved the submitted version.
